# Atypical pattern of ocular toxoplasmosis: recurrent inner foveal toxoplasmic retinitis (rifter)

**DOI:** 10.1186/s40942-023-00510-8

**Published:** 2023-11-30

**Authors:** Guilherme Macedo Souza, Laurentino Biccas, André Maia, Claudio Silveira, Rubens Belfort

**Affiliations:** 1 Department of Ophthalmology and Visual Science, Federal University of Sao Paulo, Sao Paulo, SP Brazil; 2 Instituto Paulista de Estudos e Pesquisas em Oftalmologia, IPEPO, Vision Institute, Sao Paulo, SP Brazil; 3 Ocular Oftalmologia, Vitoria, ES Brazil; 4 Clínica Silveira, Erechim, RS Brazil

**Keywords:** Uveitis, Ocular toxoplasmosis, Toxoplasma Retinochoroiditis, Posterior Uveitis, Toxoplasmosis

## Abstract

Failure to recognize the different possible clinical presentations of ocular toxoplasmosis may delay diagnosis and treatment, compromising visual prognosis. The aim of this paper is to describe an atypical pattern of ocular toxoplasmosis, not yet described. Five Brazilian patients, from 4 different referral centers, presented similar atypical pattern of ocular toxoplasmosis characterized by mild vitritis, foveal cavitation involving predominantly all retinal layers associated with adjacent inner retinal necrosis (a necrotizing retinitis with a persisting inner retinal tissue bridge and loss of subjacent retinal layers). The appearance of the OCT image resembling a “rift”, led the authors to define this pattern as a Recurrent Inner Foveal Toxoplasmic Retinitis (RIFTER), which can be considered as a new description of an atypical pattern of toxoplasma retinochoroiditis, and clinicians should be aware of it and consider testing for toxoplasmosis in patients with similar findings.

**Supplementary Information:**

The online version contains supplementary material available at 10.1186/s40942-023-00510-8.

## Introduction

*Toxoplasma gondii* is a protozoan that establishes a complex relationship with the eye, inducing variable initial retinal lesions, manipulating the immune response and further recurrences [1].

Its diagnosis primarily relies on the recognition of the clinical manifestations (focal necrotizing retinitis with secondary choroiditis adjacent to a scar, frequently associated with retinal vasculitis and vitritis), the presence of specific antibodies against Toxoplasma and exclusion of differential diagnosis [2].

Nonetheless, there are various atypical clinical presentations that may be unfamiliar to ophthalmologists, delaying both diagnosis and treatment. The atypical patterns most reported are punctate outer retinal toxoplasmosis, neuroretinitis, pseudo-multiple retinochoroiditis, pigmentary retinopathy, retinal vascular occlusion, scleritis and serous retinal detachment [3, 4].

Compared to classic retinochoroiditis seen in ocular toxoplasmosis, atypical presentations are not necessarily more severe [3].

The aim of this article is to report five similar cases of an unusual presentation of ocular toxoplasmosis.

## Case reports

### Case 1

**Fig. 1:**
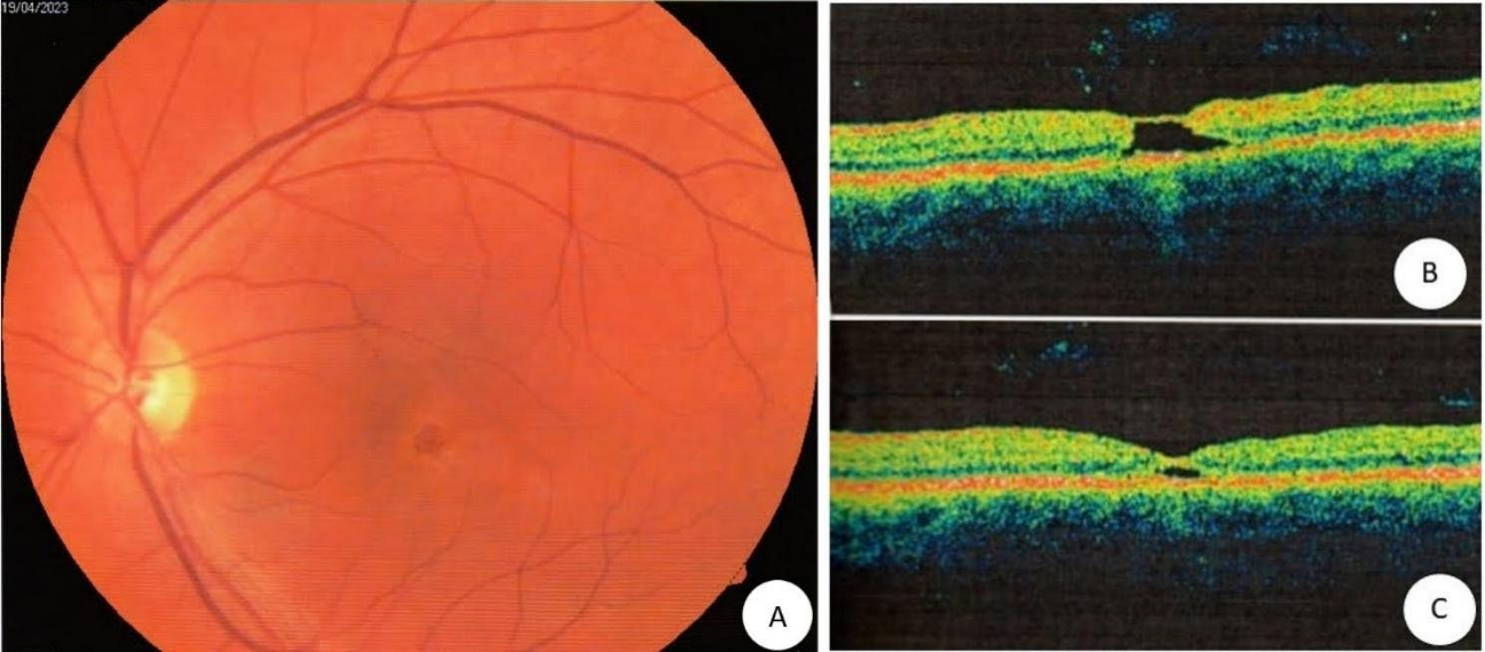
**(A)** Retinography of the left eye before treatment, showing the parafoveal yellowish ring; **(B)** Macular OCT of the left eye before treatment; **(C)** Macular OCT after after 4 weeks of treatment

A 46-year-old female patient, with unremarkable past medical history, complaining of sudden unilateral vision loss presented with a best corrected visual acuity of 20/20 in the OD and 20/100 in the OS. Exam of the right eye was normal, while the left eye showed vitreous cells and a parafoveal yellowish ring image. OCT demonstrated a subfoveal cavitation encompassing all retinal layers (Fig. 1).

Laboratory exams revealed repeated positive IgM and IgG serologies for toxoplasmosis Work up negative for other causes of uveitis.

The patient was treated with sulfamethoxazole-trimethoprim, pyrimethamine, folinic acid and oral corticosteroids, resulting in significant improvement in visual acuity to 20/25 and in the fundus aspect within four weeks.

### Case 2

An 84-year-old male patient, with a history of congenital cataract and amblyopia in the OS (best corrected vision of 20/400) and untreated systemic toxoplasmosis 4 years prior, presented with history of 15 days of a sudden vision loss in the OD. He was diagnosed as having a toxoplasmic retinochoroiditis and treated with systemic CoTrimoxazole, that was withdrawn in seven days because of acute renal failure.

**Fig. 2:**
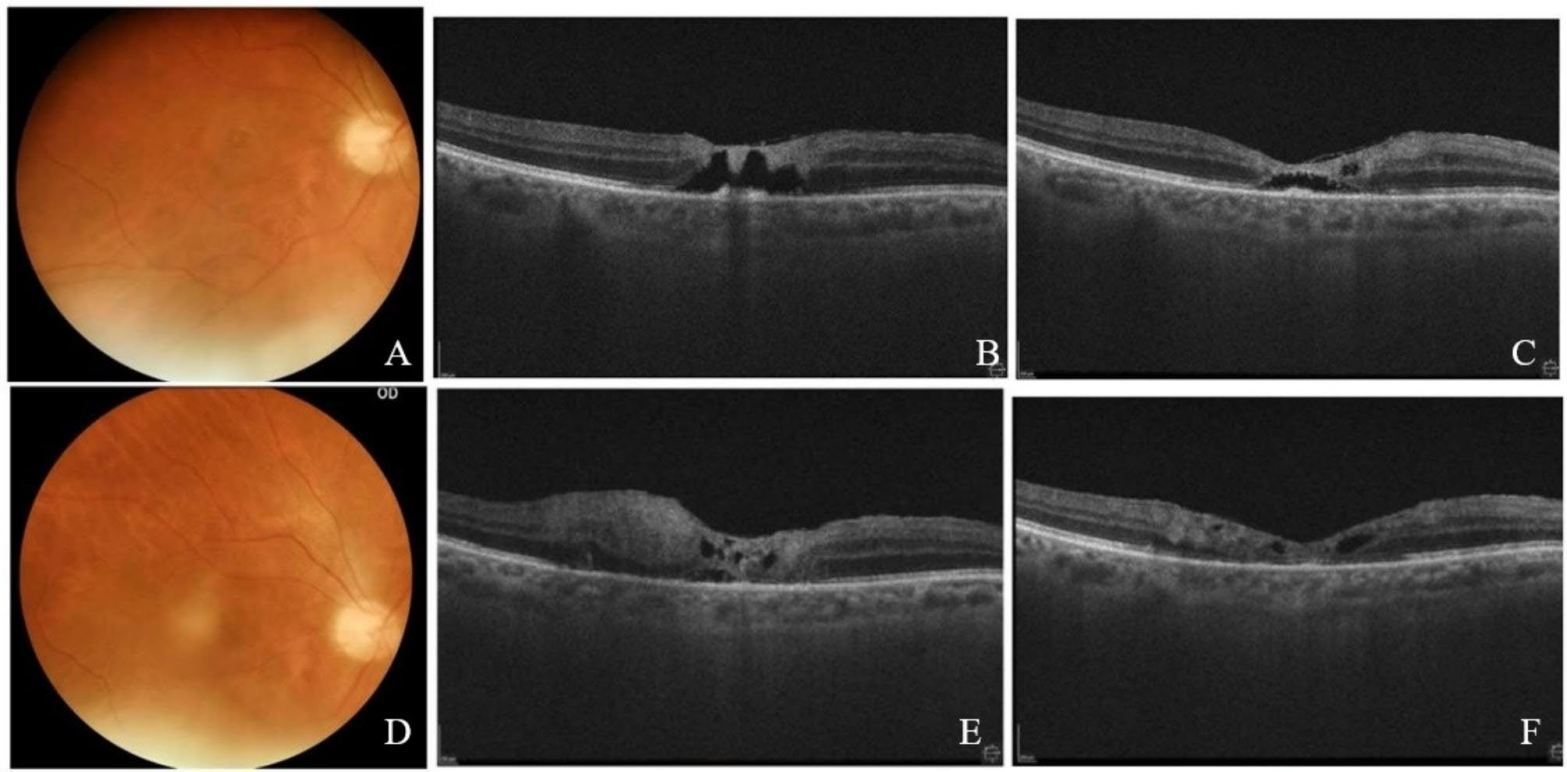
**(A)** Right eye at first presentation, showing the parafoveal yellowish ring-shaped lesion; **(B)** Macular OCT, showing the inner foveal retinitis and large cavitation; **(C)** Macular OCT of the right eye after 1 week of intravitreal clindamycin; **(D, E)** Retinography and Macular OCT at the first recurrence (two months after first presentation), showing a classic clinical presentation of ocular toxoplasmosis; **(F)** Macular OCT of the right eye after 1 week of intravitreal clindamycin to treat the 1rst recurrence

The ophthalmologic examination of the affected eye revealed anterior chamber reaction, vitreous cells and a parafoveal yellowish ring image on fundus exam (Fig. 2A). The OCT demonstrated a subfoveal cavitation encompassing all retinal layers, with a hyperreflective triangular lesion above the hyporeflective area of the cavitation, which seemed to be a necrotizing retinitis involving the inner layers of the retina (Fig. 2B). Laboratory work-up confirmed IgG reactivity for toxoplasmosis (IgM was non-reactive). Work up for syphilis and other causes of uveitis was negative. He was treated with intravitreal clindamycin (1.0 mg), with initial improvement of the vision and the fundus lesions (Fig. 2C). He evolved with subsequent 4 episodes of recurrence within 20 months, all treated with intravitreal clindamycin. Figure 2D and E shows a classic toxoplasmic lesion, presented at the first recurrence (two months after first presentation). Figure 2 F shows the OCT 1 week after intravitreal clindamycin to treat the first recurrence. At the time of the fourth recurrence, a vitreous PCR assay was positive for *Toxoplasma* and ruled out Herpesvirus.

### Case 3

**Fig. 3:**
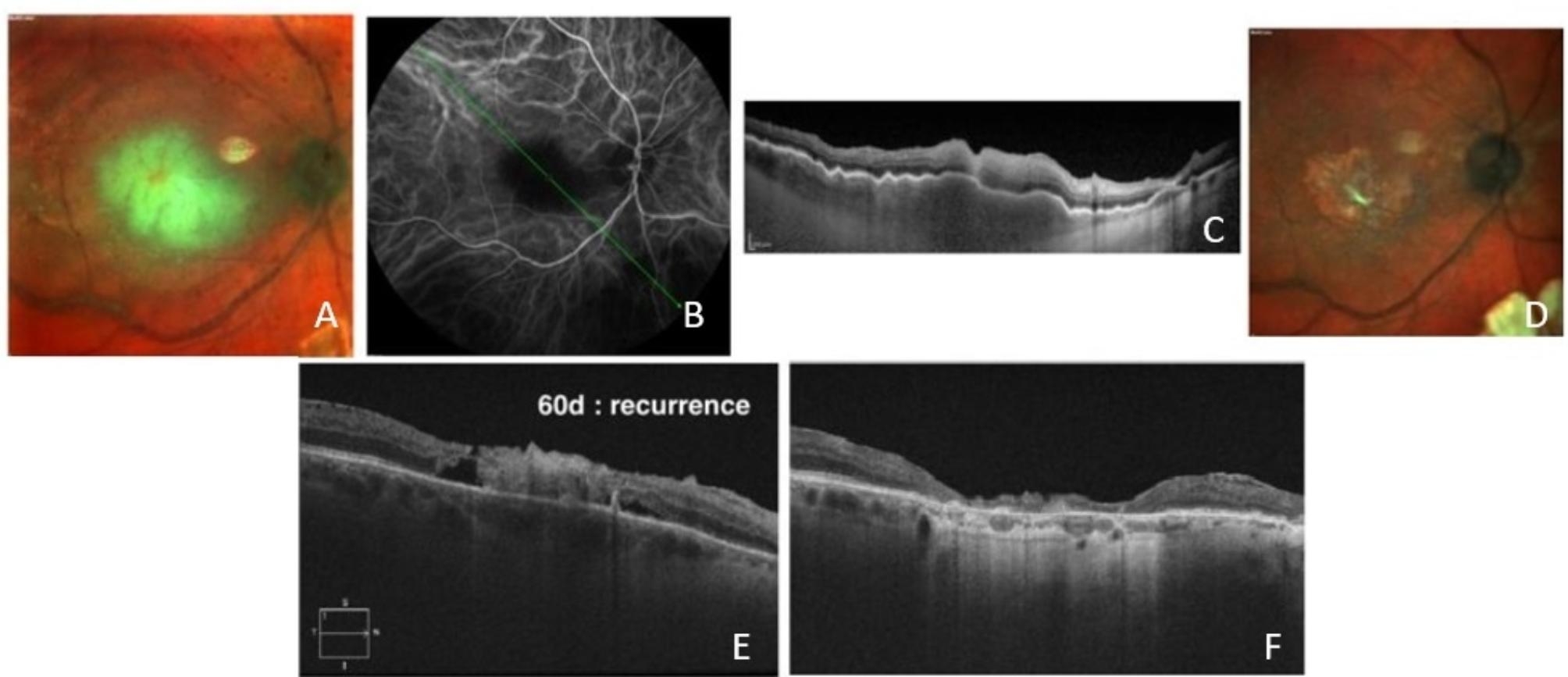
(**A**, **B** and **C**) Retinography, Fluorescein Angiography and Macular OCT of the right eye showing toxoplasmic retinochoroiditis; **(D)** Retinography of the right eye after treatment; **(E)** Macular OCT of the right eye showing recurrence of the lesion, presenting inner retinitis and foveal large cavitation; **(F)** Macular OCT after recurrence treatment

An 86-year-old Brazilian female patient, complained of sudden decrease of vision in the OD. Toxoplasmic retinochoroiditis was diagnosed (through classic clinical presentation and reactive IgG and IgM serologies) and treated with intravitreal clindamycin with clinical improvement. After 2 months, the patient experienced a recurrence, with vitreous cells and a subfoveal large cavitation and adjacent hyperintensity of the inner retina, consistent with necrotizing retinitis. This recurrence was treated with intravitreal clindamycin with resolution of the inflammatory lesions (Fig. 3).

### Case 4

**Fig. 4:**
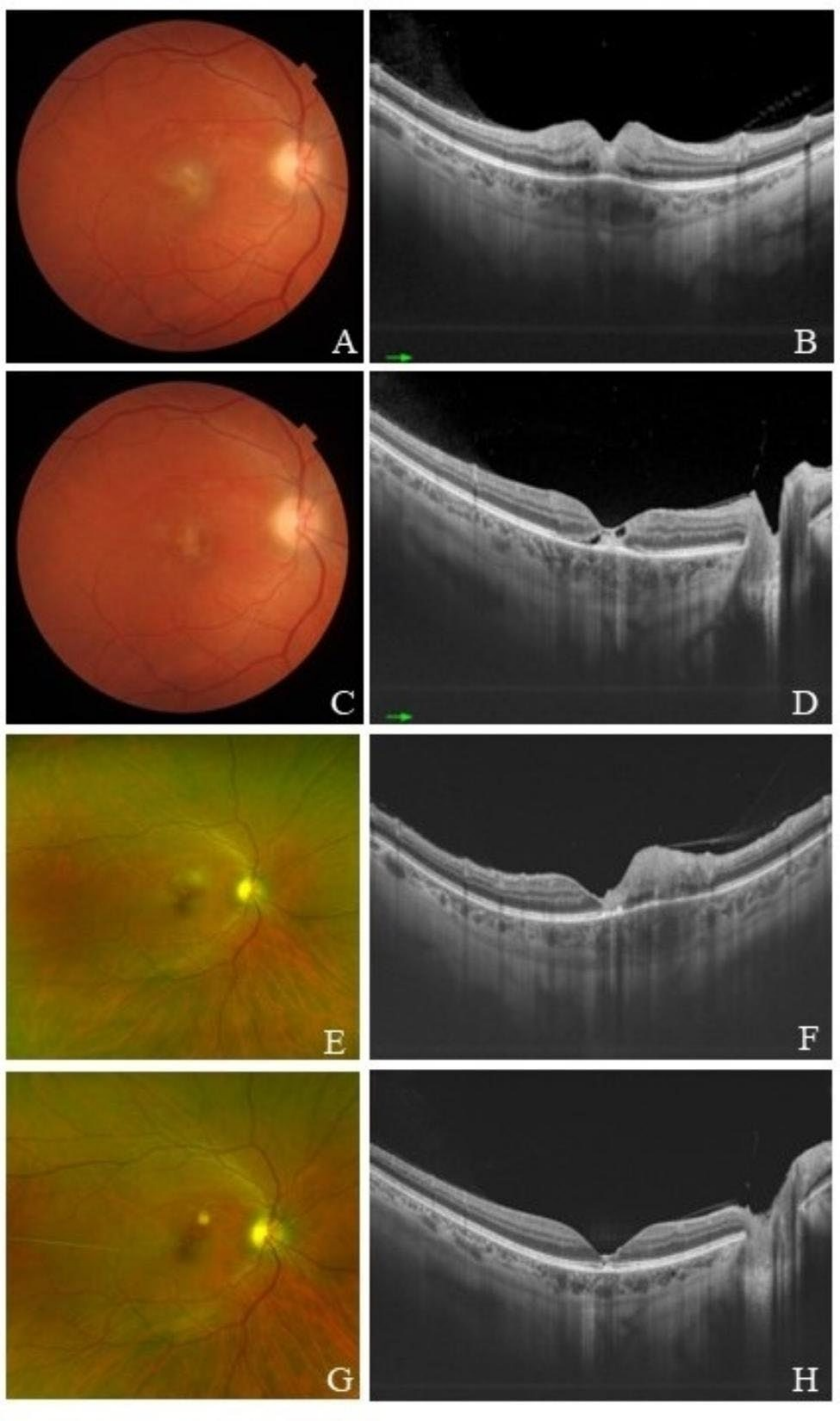
(**A** and **B**): Retinograpy and Macular OCT of the right eye at the first presentation of toxoplasmic retinitis; (**C** and **D**): Retinography and Macular OCT after treatment of the first presentation; (**E** and **F**): Retinography and Macular OCT during the ocular toxoplasmosis recurrence; (**G** and **H**): Retinography and Macular OCT after treatment of the recurrence

Female patient with 35 years old complaining of blurry vision on right eye for one week. She presented visual acuity of 20/100 OD and 20/20 OS, fundus exam with vitreous cells and exudative focal ring-shaped macular lesion on right eye (Fig. 4A and B) and positive IgG and IgM serologies for toxoplasmosis. Treatment was made with sulfadiazine and pyrimetamine with improvement (evolved with vision of 20/40 OD), and OCT after treatment presented disruption and cavitation encompassing retinal layers (Fig. 4C and D).

Two years later, the patient presented recurrence, with visual acuity of 20/50 (Figure 4E and 4F). At this point, HIV serology was positive, and started antiretroviral drugs. The treatment for the uveitis was made with sulfamethoxazole-trimethoprim, 20/50 vision OD was maintained (Figure 4G and 4H).

### Case 5

**Fig. 5:**
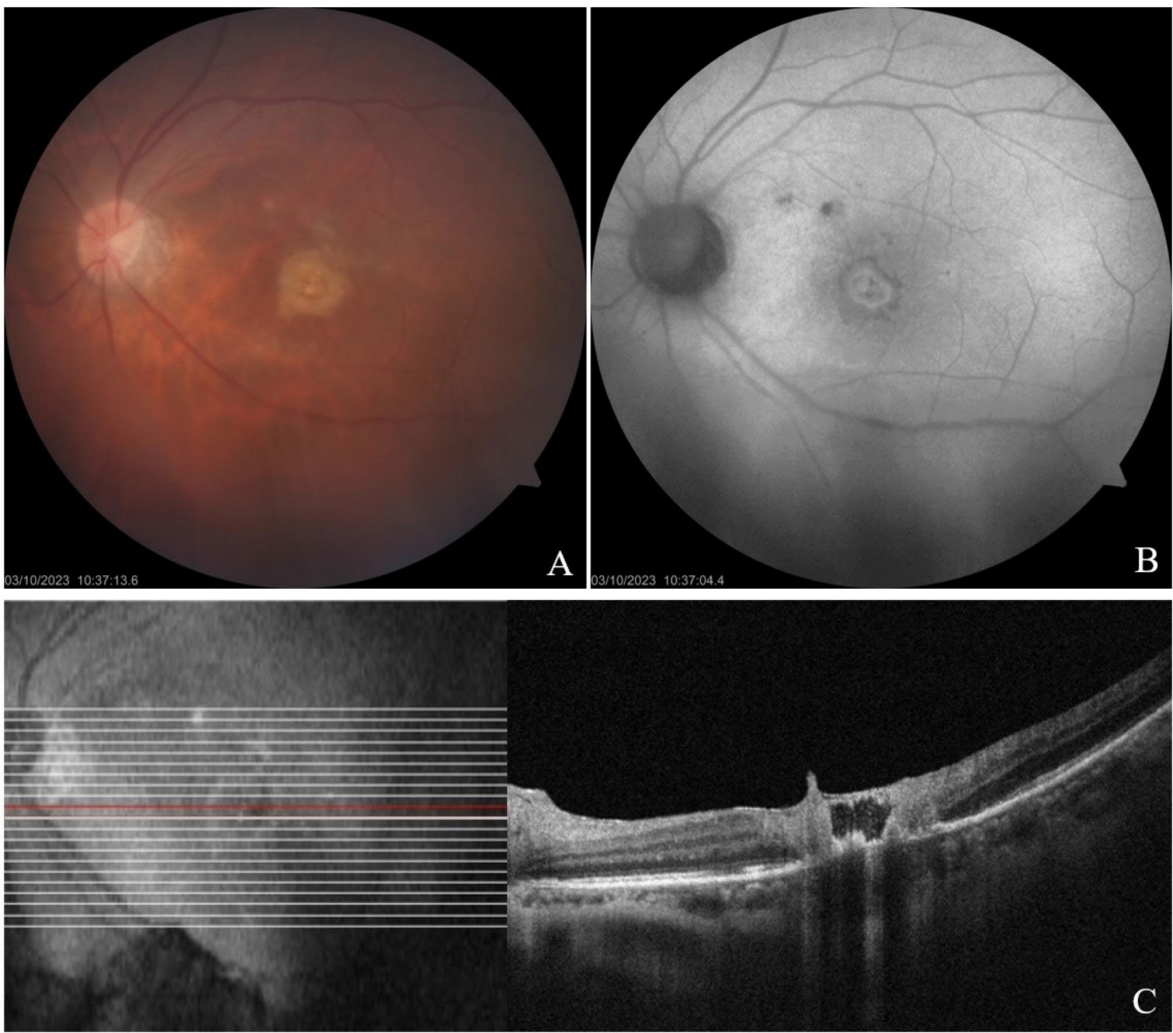
**(A)** Retinography of the left eye **(B)** Autofluorescence of the left eye; **(C)** Macular OCT of the left eye

Fifty-one-year-old hypertensive female complaining of low vision and central scotoma OS for 4 months. BCVA was 20/20 OD and counting fingers at 1 m OS. Exam of the right eye was normal, while the left eye showed vitreous cells, mild vitreous haze, and a yellowish foveal ring-shaped lesion without exudative appearance (Fig. 5A and B). OCT OS demonstrated a subfoveal cavitation encompassing retinal layers, with a hyperreflective appearance of the adjacent retina (Fig. 5). Laboratory work-up confirmed IgG reactivity for toxoplasmosis (IgM was non-reactive). Work up for syphilis and other causes of uveitis was negative.

## Discussion

All the patients described presented mild vitritis, macular involvement and foveal cavitation predominantly involving all retinal layers, which appears to be a persisting inner retinal tissue bridge and loss of outer layers, associated with adjacent inner retinal necrosis, with scant residual retinal lesions on fundus exam after resolution.

The patients 1, 2, 4 and 5 presented a yellowish ring-shaped retinal lesion on fundus exam, which was not seen in patient 3 possibly because of the adjacent previous scar tissue.

The appearance of the OCT image resembling a “rift” (which is similar to an inner limiting membrane drape sign, most commonly described in patients with macular telangiectasia type 2) [5], and the clinical pattern presentation was coined by the authors as a Recurrent Inner Foveal Toxoplasmic Retinitis (RIFTER).

These lesions were present in patients with distinct ages presenting both positive and negative IgM serology for toxoplasmosis.

The first and fifth case did not evolve with recurrence possibly due to the short follow up.

This unusual clinical presentation may represent an atypical pattern of ocular toxoplasmosis that should be recognized for early diagnosis and initiation of therapy.

Similar pattern has been reported as unilateral acute idiopathic maculopathy (UAIM) and as a near full thickness macular hole with intact overlying inner limiting membrane in cases of acquired toxoplasma retinitis [6, 7].

Clinicians should be aware of this presentation and consider testing for toxoplasmosis in patients with similar clinical findings.

### Electronic supplementary material

Below is the link to the electronic supplementary material.

Supplementary Material 1

## Data Availability

All data generated during this study are included in this published article.
