# Supplementary material for: Atypical pattern of ocular toxoplasmosis: recurrent inner foveal toxoplasmic retinitis (rifter)
Source: Int J Retina Vitreous. 2023 Nov 30;9:75. doi: 10.1186/s40942-023-00510-8 (PMC10691084; doi:10.1186/s40942-023-00510-8)
Supplement: Supplementary file 1 — Supplementary Material 1 [file 40942_2023_510_MOESM1_ESM.docx]

Federal University of Sao Paulo

Sao Paulo – Brazil

+55 11 5085-2010

Antonio Marcelo Barbante Casella, MD and Gustavo Barreto de Melo, MD

Editors-in-Chief

International Journal of Retina and Vitreous

October 04, 2023

Dear Dr. Antonio Marcelo Barbante Casella and Dr. Gustavo Barreto de Melo

We wish to submit an original article entitled “Atypical pattern of ocular toxoplasmosis: Recurrent Inner Foveal Toxoplasmic Retinitis (RIFTER)” for consideration by International Journal of Retina and Vitreous.

In this paper, we present five cases of an atypical presentation of ocular toxoplasmosis that has not yet been described.

The authors declare no issues relation to journal policies and no potential competing interests. All authors have approved the manuscript submission.

The content of the manuscript has not been published or submitted for publication elsewhere.

Sincerely,

Guilherme Macedo Souza, MD

Department of Ophthalmology and Visual Science of Federal University of Sao Paulo

gmacedo4@gmail.com
